# Population fluctuations and spatial synchrony in an arboreal rodent

**DOI:** 10.1007/s00442-019-04537-3

**Published:** 2019-10-30

**Authors:** Vesa Selonen, Jaanus Remm, Ilpo K. Hanski, Heikki Henttonen, Otso Huitu, Maarit Jokinen, Erkki Korpimäki, Antero Mäkelä, Risto Sulkava, Ralf Wistbacka

**Affiliations:** 1 Department of Biology, Section of Ecology, University of Turku, 20014 Turku, Finland; 2 Kirkkotie 127A, 02570 Siuntio kk, Finland; 3 Natural Resources Institute Finland, P.O. Box 2, 00791 Helsinki, Finland; 4 Department of Biosciences, University of Helsinki, 00014 Helsinki, Finland; 5 Linnantie 10, 63350 Sulkavankylä, Finland; 6 University of Eastern Finland, Joensuu, Savonrannantie 12a, 79940 Vihtari, Finland; 7 Department of Biology, University of Oulu, 90014 Oulu, Finland; 8 Department of Zoology, Institute of Ecology and Earth Sciences, University of Tartu, Vanemuise 46, 51014 Tartu, Estonia

**Keywords:** Climate change, Dispersal, Resource pulse, Population dynamics, Reproductive success, Squirrel

## Abstract

Climatic conditions, trophic links between species and dispersal may induce spatial synchrony in population fluctuations. Spatial synchrony increases the extinction risk of populations and, thus, it is important to understand how synchrony-inducing mechanisms affect populations already threatened by habitat loss and climate change. For many species, it is unclear how population fluctuations vary over time and space, and what factors potentially drive this variation. In this study, we focus on factors determining population fluctuations and spatial synchrony in the Siberian flying squirrel, *Pteromys volans*, using long-term monitoring data from 16 Finnish populations located 2–400 km apart. We found an indication of synchronous population dynamics on a large scale in flying squirrels. However, the synchrony was not found to be clearly related to distance between study sites because the populations seemed to be strongly affected by small-scale local factors. The regularity of population fluctuations varied over time. The fluctuations were linked to changes in winter precipitation, which has previously been linked to the reproductive success of flying squirrels. Food abundance (tree mast) and predator abundance were not related to population fluctuations in this study. We conclude that spatial synchrony was not unequivocally related to distance in flying squirrels, as has been observed in earlier studies for more abundant rodent species. Our study also emphasises the role of climate in population fluctuations and the synchrony of the species.

**Electronic supplementary material:**

The online version of this article (10.1007/s00442-019-04537-3) contains supplementary material, which is available to authorized users.

## Introduction

Fluctuations in population densities may be spatially correlated due to climatic conditions, trophic links between species and dispersal (Moran 1953; Liebhold et al. 2004). Such spatial synchrony is observed in a wide variety of organisms across distances of up to several hundred kilometres (Moran 1953; Hanski and Woiwod 1993; Sinclair et al. 1993; Ranta et al. 1995; Paradis et al. 2000). To better understand what spatial synchrony can tell us about population dynamics of a species, we need more information on the drivers and structure of the synchrony (Walter et al. 2017). The strength of synchrony may vary over both space and time, and therefore, understanding the factors behind variation in synchrony and population fluctuations may help in the management of populations in a changing world (Ranta et al. 1998; Sheppard et al. 2015; Walter et al. 2017).

Variations in weather are commonly implicated as a mechanism causing spatial synchrony in population fluctuations (Moran 1953; Hanski and Woiwod 1993; Lindström et al. 1996; Paradis et al. 2000). For example, the spatial synchrony of vole population cycles, which may span distances of over a few hundred kilometres (Sundell et al. 2004), is linked to weather conditions in winter (Huitu et al. 2008; Terraube et al. 2015). Similarly, changes in North Atlantic Oscillation (in insects; Sheppard et al. 2015) or temperature (in birds; Koenig and Liebhold 2016) are found to affect spatial synchrony. Due to ongoing climate change, weather is a particularly interesting possible force behind synchrony and population fluctuations in general (Post and Forchhammer 2002). At high latitudes, climates are changing rapidly, and understanding how weather shapes population dynamics in these regions may help predict population trajectories in the future (Ruckstuhl et al. 2008).

The effects of large-scale climatic variations in spatial synchrony may also be mediated in a bottom-up manner by trophic interactions (Liebhold et al. 2004), especially in systems with pulsed resources, such as mast seeding by trees (Ostfeld and Keesing 2000; Bogdziewicz et al. 2016). Tree mast is typically spatially autocorrelated across distances of a few hundred kilometres due to spatially correlated weather phenomena (e.g. Ranta et al. 2010; Zamorano et al. 2018). This can result in high spatial synchrony of population dynamics in consumers of the tree mast (Haynes et al. 2009; Bogdziewicz et al. 2016). For example, in the European red squirrel, population dynamics are tightly linked to the coniferous seed mast in different forest habitats (Wauters et al. 2004, 2008; Selonen et al. 2015; Turkia et al. 2018a). Similarly, in Nearctic temperate forests, white footed mouse populations fluctuate synchronously over several hundreds of kilometres, following variations in the main food sources of the mice (Haynes et al. 2009). In addition to plant–herbivore interactions, other trophic links between species, such as between predator and prey, are potential sources for spatial synchrony (Ydenberg 1987; Ims and Andreassen 2000; Liebhold et al. 2004; Haynes et al. 2009).

In species with limited movement abilities and a relatively broad degree of spatial synchrony, such as many rodent populations, dispersal is not considered to be the primary cause of spatial synchrony (Sundell et al. 2004; Ims and Andreassen 2005; Huitu et al. 2008). For example, lifetime dispersal distances of the Siberian flying squirrel, *Pteromys volans*, are short, on average 1–2 km (Hanski and Selonen 2009; Selonen et al. 2010b; Selonen and Wistbacka 2017), and are insufficient for synchronising population dynamics of adjacent populations based on the comparison of demography of two populations (Brommer et al. 2017). Both climate (Huitu et al. 2008) and food (Haynes et al. 2009) are linked to spatial population synchrony in rodents. However, the direct effect of climate, while controlling the effect of food on population fluctuations and spatial synchrony, has been rarely studied in mammals.

We studied factors influencing population fluctuations and spatial synchrony in fluctuations in the Siberian flying squirrel in Finland, using data on long-term occupancy rates of nest-boxes or forest patches as indices of population abundance. The flying squirrel differs from rodent species previously studied for spatial synchrony, such as voles, in that flying squirrels are less abundant in numbers and are protected species (Selonen and Mäkeläinen 2017). Previous studies indicate that both climate and winter food (mast of deciduous trees) influence the reproduction and life-time reproductive success of female flying squirrels (Selonen et al. 2016; Selonen and Wistbacka 2016; Hoset et al. 2017). Thus, climate and tree mast potentially synchronise population fluctuations over distances equivalent to those observed in populations of voles and mice (Sundell et al. 2004; Huitu et al. 2008; Haynes et al. 2009). Specifically, increased precipitation in winter has been observed to have positive effects on reproduction in flying squirrels (Selonen et al. 2016; Selonen and Wistbacka 2016). This somewhat surprising finding may be related to improved plant growth in spring (flying squirrels eat buds and leaves in spring and summer). Tree mast in winter has also been observed to be more important than forest structure for lifetime reproductive success in flying squirrels (Hoset et al. 2017). Previous studies indicate that predators are not likely to drive population fluctuations of flying squirrels (Koskimäki et al. 2014, but see Jokinen et al. 2019). Nevertheless, in our analyses we control for potential effects of main predators by utilising abundance indices of pine marten (*Martes martes*) and vole population sizes as a proxy for the breeding success of vole-eating owls (Korpimäki and Hakkarainen 2012). Vole population size is tightly linked with the breeding density of owls in Finland (Korpimäki and Sulkava 1987; Sundell et al. 2004), which is positively associated with owl predation pressure on flying squirrels (Selonen et al. 2010a).

A prerequisite for large-scale spatial synchrony is that populations are affected by factors that operate across the area. Thus, we begin our analysis (1) by correlating the growth rate of flying squirrel populations with climatic variables and tree mast. We predict that (a) a warm autumn and winter positively contribute to population growth detected next spring due to enhanced juvenile survival; instead (b) increased precipitation in winter increases population growth with 1 year lack due to positive effect on reproduction (Selonen and Wistbacka 2016). We further predict that (c) tree mast is positively related to population growth in the following year (see Selonen and Wistbacka 2016). We analyse (2) whether population fluctuations are synchronised over large distances (up to 400 kilometres) and whether the synchrony depends on distance between populations. This is analysed by spatial autocorrelation in population growth rate and abundance. Finally, (3) we perform a wavelet analysis to study whether there is cyclicity in population fluctuations or changes in population fluctuations across the study period.

## Materials and methods

### Study species

The flying squirrel is an arboreal rodent inhabiting the boreal zone from eastern Siberia and Japan to north-eastern Europe, where the species is found in Finland and Estonia (Selonen and Mäkeläinen 2017). The species is mainly associated with spruce-dominated mixed forests containing deciduous trees for foraging and nesting. Flying squirrels nest in tree cavities, nest-boxes and dreys. The mating season starts in mid-March (Selonen and Mäkeläinen 2017). After the first litter is born in April, the females can sometimes have a second litter born in June, especially when food availability is high (tree mast preceding reproduction, Selonen and Wistbacka 2016). Juveniles disperse in the late summer of the year of birth (Hanski and Selonen 2009; Selonen et al. 2010b). Females are territorial, living in non-overlapping home ranges (on average 7 ha) indicating that competition for nesting sites occurs, while males live in overlapping home ranges (on average 60 ha) encompassing several males and females (Selonen et al. 2013). Breeding dispersal is rare, but natal dispersal distances are usually 1–2 km (Hanski and Selonen 2009; Selonen and Wistbacka 2017). Most individuals live only 1 or 2 years (those that survive the natal period), but individuals as old as 7 + years have been recorded (Hoset et al. 2017). The main predators of flying squirrels are large owls, such as the Ural owl (*Strix uralensis*), the goshawk (*Accipiter gentilis*) and the pine marten, but flying squirrels are not the main prey of any predators (Selonen et al. 2010a; Selonen and Mäkeläinen 2017).

### Population monitoring data

We used long-term monitoring data from nine different flying squirrel populations, spanning an average of 16 ± 6 years (± sd; range 5–25 years; Table 1). Four of the populations were divided into smaller parts (see below) resulting in a total of 16 time-series used in this paper. Seven of the nine surveys were on flying squirrel populations living in nest-boxes (for nest(-box) use of the flying squirrel, see Selonen and Mäkeläinen 2017). Nest-boxes were checked in late spring–early summer at each study site during single nest-box checking session, presence of flying squirrel individual indicating occupancy of the nest-box. Nest-boxes were placed in separate forest patches one box in each patch in most cases. However, in Vaasa and Kauhava study areas they were in groups of 2–4 nest-boxes, but in these cases the group of nest-boxes within a forest patch was treated as a sampling unit. Two of the data sets were surveys of occupancy based on the presence of faecal pellets of the species. Detecting pellet presence is a commonly adopted protocol for surveying flying squirrels (see e.g. Mönkkönen et al. 1997; Hurme et al. 2008; Remm et al. 2017). Pellets are relatively easy to find due to their yellow colour and deposition location (usually at the base of large aspen and spruce trees), therefore detection probability can be assumed to be high and the risk of false absences low (Hurme et al. 2008). The survey unit in these data was a forest patch (see Table 1 for number of surveyed patches). The forest patch was surveyed ones in late winter/early spring when faeces dropped during the winter can be detected from the ground near trees. The survey method was unchanged across years and based on search from the total area of the forest patch, search stopping if pellets were detected (see e.g. Mönkkönen et al. 1997; Hurme et al. 2008; Remm et al. 2017). The sampling unit (forest patch) was quite similar for both the nest-box and pellet surveys, although the method to detect presence varied. The detection probability is significantly lower in nest-box surveys than in pellet surveys (see results), but we controlled for study site and used growth rate or study site-specific change in abundance in our analysis. Thus, the variation in detection probability should not affect our results. Indeed, when adding survey method as a factor to our models exploring variation in growth rates (see below), its effect was statistically not significant; hence we are confident it did not affect our growth rate estimates. The time-series were made up from the yearly occupancy rate in surveyed populations (Supplement Fig. S1). That is, the yearly variation in (nest-boxes used)/(all surveyed nest-boxes) or (forest patches occupied by the flying squirrel)/(all forest patches surveyed in the study area).

**Table 1:** Description of long-term data sets and study sites used in the analysis of flying squirrel population fluctuations and spatial synchrony

Study site	Coordinates	Years studied	Size of study area (km^2^)	Flying squirrels observed yearly (occupancy rate)	Total no. checked^a^	References
Alavus	62.801°N 23.577°E	1995–2011	45	15 ± 8 (0.12 ± 0.06)	130 ± 27 nest-boxes	Koskimäki et al. (2014)
Anjalankoski	60.724°N 26.982°E	1999–2005	33	12 ± 6 (0.08 ± 0.05)	161 ± 20 nest-boxes	Hanski (2006)
Kauhava	63.091°N 23.017°E	2002–2015	1300	35 ± 17 (0.1 ± 0.03)	357 ± 129 nest-boxes	Turkia et al. (2018b)
Luoto^b^	63.809°N 22.785°E	1993–2011	25	35 ± 12 (0.18 ± 0.05)	189 ± 40 nest-boxes	Brommer et al. (2017)
Vaasa^b^	63.045°N 21.654°E	1992–2014	25	32 ± 17 (0.16 ± 0.05)	211 ± 107 nest-boxes	Lampila et al. (2009), Brommer et al. (2017)
Mynämäki	60.664°N 22.194°E	1992–2003	300	5 ± 2 (0.05 ± 0.02)	113 ± 24 nest-boxes	Vesa Sarola, unpublished
Sauvo	60.341°N 22.723°E	1992–2003	300	8 ± 3 (0.08 ± 0.03)	96 ± 22 nest-boxes	Vesa Sarola, unpublished
Muurla-Lohja^b^	60.358°N 23.705°E	2001–2011	700	41 ± 7 (0.59 ± 0.08)	69 ± 5 forest patches	Pimenoff and Vuorinen (2002–2012)
Virrat-Keuruu^b^	62.322°N 24.119°E	1988–2012	300	49 ± 14 (0.6 ± 0.07)	82 ± 20 forest patches	Sulkava and Sulkava, unpublished

^a^Number of nest-boxes or forest patches for which assessment of flying squirrel presence is based on individual presence or on faecal pellet presence, respectively

^b^For spatial synchrony analysis, the study area was divided to smaller parts

The average distance between two study sites was 193 ± 121 km (minimum 2 km, maximum 409 km; based on the geographic centre of each study site and including the divided study sites, see below). All study sites were in typical Finnish forest-dominated rural landscapes, where pine and spruce-dominated forest cover made up almost two-thirds of the landscape, and the rest was made up of agricultural areas, human settlements and water. We were unaware of any differences between study sites in the landscape structure that would have influenced the current analysis. However, the size of the monitoring area used in different study sites varied (Table 1), pellet survey sites being larger than nest-box survey sites. However, the Kauhava nest-box study site was exceptionally large due to a lower density of nest-boxes than in the other study sites, but the study area size had no obvious effect on observed occupancy rate in our data (*F*_1,5.9_ = 53, *p* = 0.5).

**Fig. 1:**
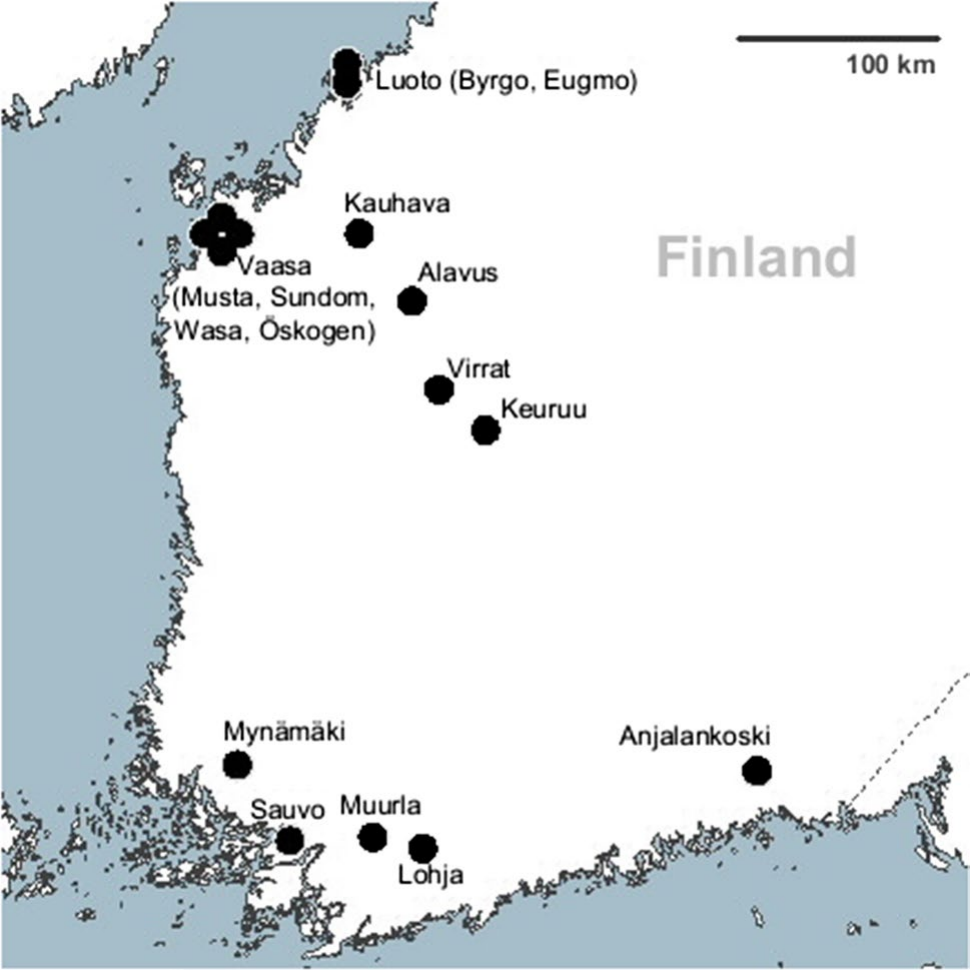
Location of flying squirrel population monitoring sites in Finland. *n* = 15 with 16 time-series; the point for the Öskogen subarea in Vaasa area represents two time-series of different periods (1992–2001 and 2001–2012) surveyed with different methods

For the spatial synchrony analysis, the four of the nine study sites listed in Table 1 were divided into smaller parts so that, in the end, we had 16 time-series: Virrat-Keuruu, Muurla-Lohja and Luoto were divided into two parts and Vaasa into four parts (see Fig. 1). In the latter two study sites, the division of the sites was done according to landscape features (open fields and other structures), which acted as barriers for dispersal of the flying squirrels living in the area (for further information on these study areas see Lampila et al. 2009; Hoset et al. 2017). The entire Vaasa area was analysed as a single time-series between 1992 and 2001 (Lampila et al. 2009). After 2001, the area was expanded and divided into four parts as described above (Musta, Sundom, Wasa and Öskogen). Thus, in the end, there were five time-series from the Vaasa area, i.e. 1 time-series during 1992–2001 (Lampila et al. 2009) and four time-series during 2001–2012 (Brommer et al. 2017), which were treated separately. The separation of the Virrat-Keuruu and Muurla-Lohja is based on placement in two municipalities with a lower sampling density on the border area. By using divided study sites, we gained data for studying synchrony within the 30 km radius of spatial autocorrelation of a population density, as defined by Remm et al. (2017). We expected spatial synchrony to be clear within this range. In the analysis for effects of climate, tree mast, and predators on the flying squirrel growth rate, we used the data of undivided study sites (*n*_sites_ = 9). Thus, the spatial autocorrelation in density (Remm et al. 2017; see above) did not have an effect in this analysis.

### Food availability data

Recent studies indicate that the availability of alder, *Alnus* sp., catkins in the winter/spring preceding reproduction is an important determinant of breeding success in flying squirrels (Selonen and Wistbacka 2016; Hoset et al. 2017). Catkin production varies considerably between years and forms a pulsed resource in the forest (Ranta et al. 2008; Selonen et al. 2016). The weather conditions during the summer preceding mast largely determine catkin production in autumn (pollen production in next spring), but trees seldom manage to produce mast for two successive years (Ranta et al. 2008). Following earlier studies (Selonen and Wistbacka 2016; Selonen et al. 2016; Hoset et al. 2017), we used aerial pollen estimates as a proxy for alder catkin production (Ranta et al. 2008; Selonen et al. 2016). Pollen data were collected in spring by the aerobiology unit of the University of Turku from 10 different locations in Finland using EU standard methods and Burkard samplers. The data consisted of accumulated sums of average daily counts of airborne pollen in 1 m^3^ of air during spring (Ranta et al. 2005). The closest sampling site was used from each study site, this being 0–90 km from the study sites. The spatial autocorrelation in catkin production is high, up to a distance of 500 km (Ranta et al. 2008; Zamorano et al. 2018). Consequently, our index was not exact, but it could describe the yearly variation in catkin production in our study sites. We also tried using birch-catkin data, another tree species used by the flying squirrel, in our analysis (data collected by Finnish Forest Research Institute; see Selonen et al. 2016). However, birch mast correlates with alder mast and we selected the one with lower AIC value to be used in the current analysis (alder was better fitted to data and was selected to the analysis).

### Weather indices

For each study site, we used mean monthly weather information from the closest weather station maintained by the Finnish Meteorological Institute (Venäläinen et al. 2005). Weather recording stations were at the same altitude as the study sites and located within 10 km from our study sites. At these distances, there is minimal spatial variation in mean monthly weather measures in Finland.

We counted weather indices for the following periods: For winter weather, we used mean temperature, precipitation and depth of snow cover in December–February (based on the mean of each month). Precipitation in winter can be either snow or rain. For spring weather, we used mean temperature and precipitation in April–May. Summer weather was calculated as the mean of June–August and autumn weather, the mean of October–November. March and September were excluded, as they could not be unequivocally assigned to a specific season in any of our study sites.

Weather variables were derived primarily for the year preceding flying squirrel surveys (year_*t*−1_), but in the cases of winter temperature, precipitation, and snow cover, data from the current winter (year_*t*_) were also included in our analysis. The weather variables were expected to have delayed effect on occupancy patterns following our predictions (in aims), but weather has potential to affect flying squirrels throughout the whole year. Thus, we included all seasons preceding our surveys in year_t_ and year_t-1_ to our analysis. Weather also determines tree mast available for flying squirrels, but because tree mast was included to our analysis we do not expect indirect effects of food mediated by weather. Flying squirrel population monitoring was performed yearly in late spring–early summer (nest-box surveys) or late winter–early spring (pellet surveys). Thus, the timing of surveys slightly differed (nest-box and pellet surveys), but both of the survey methods describe the occupancy status of flying squirrel territories after the winter, and before weaning of juveniles. The survey method (nest-box vs pellet survey) did not affect growth rate (*F*_1,124_ = 0.09, *p* = 0.77) and was dropped from the final model.

### Predator indices

To estimate predation pressure in our study areas, we used (a) snow-track data for the pine marten and (b) vole abundance data (index of owl activity), both collected by the Natural Resources Institute Finland (Luke). The pine marten snow-track data were obtained from the wildlife triangle censuses coordinated by Luke (Lindén et al. 1996). Wildlife triangle counts have been performed each winter in Finland since 1989 in January–March by trained volunteers. The basic unit in the scheme is an equilateral triangle with 4 km sides permanently marked in the field. On average, 830 triangles located randomly in forested areas throughout the country are counted annually. Within an area of 50 × 50 km^2^, there is an average of five triangles monitored each year (Pellikka et al. 2005). The animal abundance is reported as an index derived from the number of snow tracks observed crossing the route per 24 h of fresh snow per 10 km of census. In our analysis, we used the mean index of the municipality where the flying squirrel monitoring site is located.

The vole abundance index is obtained from biannual vole monitoring trappings carried out in ca. 20 sites throughout Finland. For this analysis, we employed data from mean of standardised abundance estimates of the 1–3 closest sites to the monitored flying squirrel sites (on average 56 ± 33 km from a flying squirrel site, which is a distance with high spatial autocorrelation in vole abundance; Sundell et al. 2004), so that we got continuous vole abundance estimates for each flying squirrel study site. Voles were trapped in both field and forest habitats at each site using ca. 100 standard metal snap traps baited with bread for one night. The vole index was calculated as: (pooled number of trapped voles of the genera Microtus and Myodes + 1)/(sum of traps set in both habitats) × 100.

### Statistics

We modelled the flying squirrel population growth rate with linear models in Proc Glimmix in SAS 9.3. Population growth rate (Gaussian distribution; we also performed the analysis using population abundance, see Table S2) was calculated as *R*_t_ = log(*N*_*t*_/*N*_*t*−1_), where *N*_*t*_ is population abundance in year_*t*_, and *N*_*t*−1_ is abundance in the previous year (Bjørnstad et al. 1999; Liebhold et al. 2004). Occupation rate (occupied/all surveyed) was used as a proxy for population abundance (*N*_*t*_ and *N*_*t*−1_). Site (*n*_sites_ = 9) was used as a class fixed variable. Alder pollen estimates, predator pressure indices, and precipitation and temperature during different seasons were included in the model, separately for year_t_ and year_*t*−1_. In addition, *N*_*t*−1_ was included in the model. Correlation between variables included to final models remained moderate (variance inflation factor < 4; Table S3).

Strength and range of spatial autocorrelation in population growth rate and abundance were analysed with general additive models (GAM). In this model, we explained the correlation between all possible pairs of study sites with the geographic distance between the sites. Pearson’s correlation coefficients (*r*) were used to calculate the pairwise correlations between growth rates or abundances of sites (*n*_sites_ = 16, i.e. four study sites were divided into separate time-series, see above). In model fitting, site pairs were weighted according to the number of sampling years the respective sites had in common. Longer time-series had a stronger impact on the GAM models. As the possible values of Pearson’s correlation coefficient are limited between − 1 and + 1, we applied a Fisher *z*-transformation before the model fitting to meet the assumption that all predicted values must lay within the range. That is, the transformed value *z* = 0.5 × ln[(1 + *r*)/(1 − *r*)], where *r* is Pearson’s correlation coefficient of a particular pair of sites (Zar 1996). For visual presentation, model predictions were back-transformed. Confidence limits and significance of the model predictions were gained iteratively using bootstrapping for two randomisation procedures (Manly 2007). First, we randomly assembled sample sets of the same size as the empirical data set without data for distance between sites. In the second randomisation procedure, random shuffling of the distance matrix was paired with the empirical matrix of correlations. In the iterations, GAM models were fitted with the same base parameters as the empirical models. In both cases, 95% confidence limits were determined by excluding 5% of extreme values from the results of simulations for each distance of site pairs. As a result, we were able to test the modelled autocorrelogram against a null model of absence of spatial autocorrelation, and against a lack of overall correlation without taking into account distance between sites. To estimate the general statistical significance of the models, we performed adjusted Mantel tests based on coefficients of determination (*p* = proportion of $r_{\text{resampled}}^{2} \, > r_{\text{empiric}}^{2}$; Manly 2007). In all cases of the iterative analysis, we used 5000 permutations. R package mgcv was used for GAM modelling (Wood 2011).

Finally, a wavelet analysis was conducted on the time-series of flying squirrel abundance. Wavelet analysis is intended to detect temporal cyclicity and changes in periodicity in time-series through the visualisation of a wavelet power spectrum (squared absolute value of the wavelet transform). The wavelet analysis expresses the amplitude of the time-series as a function of period and time (Torrence and Compo 1998). The period corresponds to the temporal lag between two sequential peaks or crashes in the time-series data. The analysis was initially carried out separately for 15 time-series (excluding the site, Anjalankoski, with time-series too short for the analysis). This resulted in 15 sets of values of a wavelet bias-corrected power spectrum (Liu et al. 2007). The spectra represent how well the functions of wavelet transform with different periods (2–8.5 years) fit the flying squirrel population abundance data between 1988 and 2015. This resulted in a two-dimensional data matrix of values of wavelet power, arranged by years in columns and wavelet period in rows. The values were normalised to a mean of zero and standard deviation of one for each site, and we calculated the mean of the normalised spectra over the different sites. The mean spectrum was tested for deviation from randomness using a 95% confidence interval of Gaussian normal distribution at every value of time and period of the pooled spectrum. An R package biwavelet was used for the wavelet analysis, using a Morlet-type wavelet transform (Gouhier et al. 2016).

## Results

The annual occupancy rate in nest-box surveys was on average 0.12 ± 0.06 (± sd) and in forest site surveys 0.60 ± 0.07 (Table 1). There was no temporal linear trend in the occupancy rate of flying squirrels (effect of year in model described in supplement Table S2: estimate − 0.001 ± 0.006, *z* = − 0.24, *p* = 0.81).

**Fig. 2:**
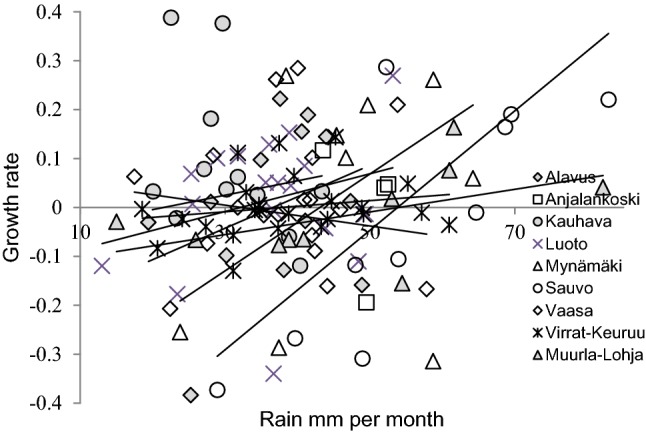
The relationship between winter rain (year_*t*−1_) and flying squirrel growth rate in nine survey sites of flying squirrels in Finland. Solid lines indicate the direction of the association in each study site

Variation in the main food abundance did not affect the growth rate in flying squirrel populations (alder pollen in Table 2), but increased precipitation in winter year_t-1_ had a clear positive effect on growth rate (Table 2; Fig. 2; the results were similar when analysed as population abundance, see Table S2). Other weather variables and predator indices had no obvious effect on growth rate, except that summer temperature had a slight positive effect (Table 2). The growth rate was strongly density-dependent (Table 2).

**Table 2:** The effects of food, predators, and weather on flying squirrel population growth rate in 9 study sites in Finland

Variable	Average ± sd	Estimate ± sd	*F*_1,104_	*p*
Previous year^a^				
Occupancy rate year_*t*−1_		− 0.93 ± 0.1	94	**0.0001**
Alder pollen	1450 ± 1700 in 1 m^3^ of air	− 0.001 ± 0.001	1.31	0.26
Marten snow track	1.1 ± 0.55 tracks in 24 h/10 km	0.03 ± 0.03	0.82	0.37
Vole index	9.4 ± 6.1 voles/100 trap nights	− 0.002 ± 0.002	0.57	0.45
Winter rain	41 ± 13 mm	0.003 ± 0.001	7.1	**0.008**
Winter snow cover	24 ± 11 cm	− 0.002 ± 0.002	1.18	0.28
Winter temperature	− 5.1 ± 2.7 °C	− 0.008 ± 0.01	0.72	0.4
Spring rain	34 ± 13 mm	− 0.001 ± 0.001	0.53	0.47
Spring temperature	5.9 ± 1.4 °C	0.002 ± 0.02	0.03	0.87
Summer rain	67 ± 21 mm	0.002 ± 0.003	0.25	0.62
Summer temperature	15.2 ± 1.2 °C	0.037 ± 0.016	5.1	**0.03**
Autumn rain	59 ± 24 mm	− 0.001 ± 0.006	0.03	0.86
Autumn temperature	4.8 ± 1.6 °C	− 0.004 ± 0.01	0.17	0.68
Current year^a^			F_1,109_	
Occupancy rate year_*t*−1_		− 0.97 ± 0.1	101	**0.0001**
Alder pollen	1450 ± 1700 in 1 m^3^ of air	0.0001 ± 0.0002	0.04	0.85
Marten snow track	1.13 ± 0.55 tracks in 24 h/10 km	− 0.04 ± 0.03	1.4	0.23
Vole index	9.7 ± 6.3 voles/100 trap nights	− 0.0004 ± 0.002	0.03	0.86
Winter rain	40 ± 13 mm	0.001 ± 0.002	0.31	0.57
Winter snow cover	25 ± 12 cm	0.001 ± 0.002	0.3	0.58
Winter temperature	− 5.2 ± 2.8 °C	0.007 ± 0.01	0.43	0.52

See “Materials and methods” section for model structure

*p* < 0.05 with bold

^a^Previous and current year values modelled in separate models, that is, in the previous model environment variables are from year_t-1_ and in current model from year_*t*_. Climate variables are averages per month; alder estimate is sum across the pollen season in spring

**Fig. 3:**
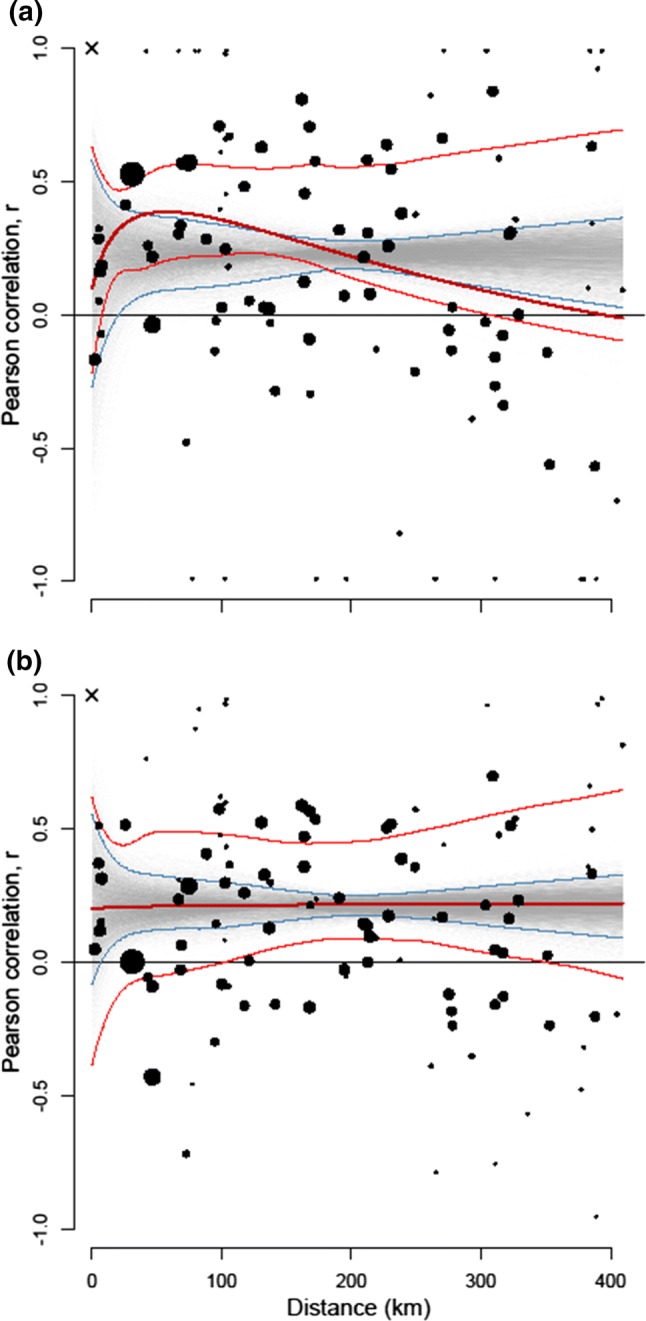
Dependence of population synchrony (pairwise correlation between time-series) on geographic distance of **a** population growth rates, and **b** abundance estimate during the whole study period from 1988 to 2015. The red lines represent the empirical GAM model and its 95% confidence interval (bootstrapped with 5000 permutations). The black dots denote the pairs of sampled sites, while dot size indicates pair weight in the model according to the number of commonly sampled years (3–25). The grey shade represents density of the GAM models of the 5000 randomly permutated data sets. The blue lines indicate 95% confidence limits of the scenario of total randomness, the null model. The *times symbol* at the upper left corner indicates the point of self-correlation that was excluded from the analysis (colour figure online)

We detected spatial synchrony in our data, i.e. according to the spatial autocorrelation analysis, the average correlations between sites are statistically significantly *r* > 0, both for growth rate and population abundance (Table 3, Fig. 3). However, the synchrony in growth rates between sites was not unequivocally related to distance between study sites (Table 3): At the closest distances the populations expressed no clear synchrony, but the average global synchrony was positive, indicating general large-scale population dynamics over the whole study area. The synchrony may decline after pairwise distances over 200 km, but, in general, the trend in synchrony was not statistically significant (Fig. 3a, Table 3).

**Table 3:** Dependence of population synchrony on geographic distance in flying squirrel time-series data

	Average synchrony, Pearson’s *r*	*N*_sites_	*N*_pairs_	GAM			p_5k permut._
				Edf	*F*	*r*_adjusted_^2^	
Abundance 1988–2015	0.18, CI 0.05–0.30	16	212	1.00	0.02	− 0.005	0.96
Growth rate 1989–2015	0.19, CI 0.02–0.34	16	212	1.89	5.57	0.04	0.07

The estimated 95% confidence intervals (CI) of the average synchrony are based on 5000 bootstrap permutations; the estimates of statistical significance of the GAMs (*p*) are based on comparisons of the empirical adjusted *r*^2^ values with 5000 randomly permuted 0-models (lack of spatial dependence)

**Fig. 4:**
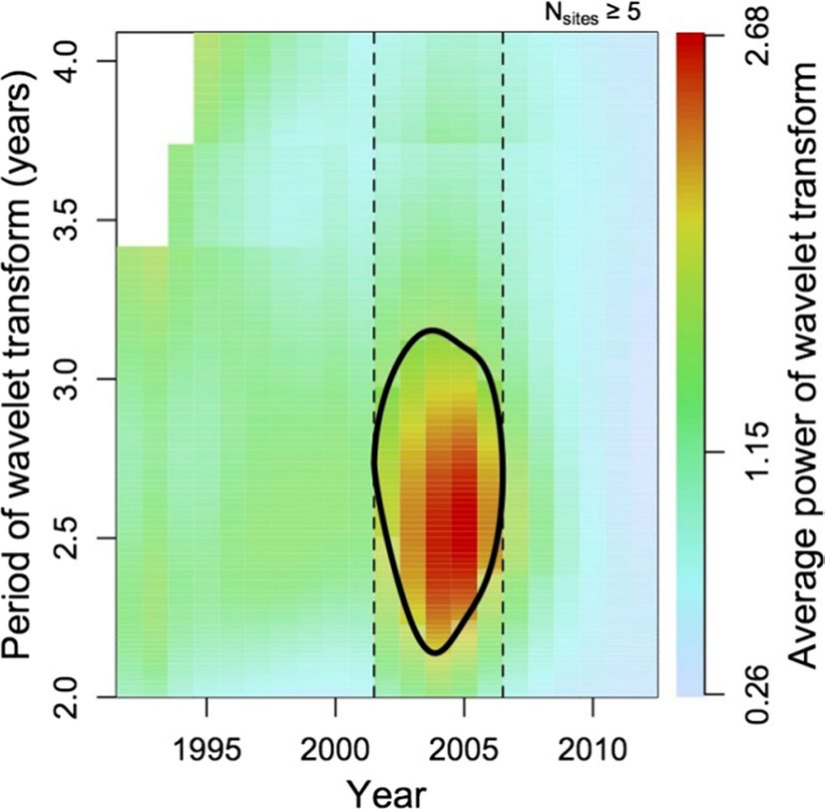
Averaged bias-corrected wavelet power spectrum (*n* = 15). The bold line delineates the area in which the mean power of the spectra is significantly higher than overall average (1.15). The vertical dotted lines delineate the years (2002–2006) when significant simultaneous cyclicity was observed. The coloured part of the diagram represents the temporal region of overlapping spectra of at least 5 study sites (max. = 14) (colour figure online)

According to the wavelet analysis, no indication of permanent cyclicity in population abundance could be detected (Fig. 4). That is, the average high-power spectrum in Fig. 4 does not cover the whole study period (1988–2015). However, there was statistically significant regularity in fluctuations with a period length of 2–3 years for years 2002 to 2006 (Fig. 4). During this period, there was less precipitation in the previous winter (year_*t*−1_) compared to that of the other study years (rain 73 ± 22 mm in years 2002–2006; of the other years: 85 ± 27 mm; *F*_1,105_ = 9.8, *p* = 0.002; other environmental variables *p* > 0.05). Wavelet analysis cannot be used to analyse synchrony in population fluctuations, but during the period with regular fluctuations, populations’ synchrony remained statistically significant for growth rate (average *r* = 0.32, Supplement Table S1), while being not statistically significant for the rest of the study period (average *r* = 0.12, Table S1). Yearly mean growth rates between study sites can be seen in supplement Fig. S2.

## Discussion

We analysed the effects of weather, tree mast and predators simultaneously on flying squirrel population fluctuations, and only precipitation in winter (year_*t*−1_) was clearly linked to flying squirrel population growth rates. There was overall spatial synchrony in population fluctuations, but it was not unequivocally related to distance between populations. Periodicity in population fluctuations was detected during a short time period.

The observed period with regular population fluctuations occurred during the longest period of years with low winter precipitation in our data set. This supports the critical role of weather on population dynamics of the species and also the possible effects of climate change, because winter weather is predicted to change rapidly in northern latitudes (Ruckstuhl et al. 2008). However, the mechanism behind observed regularity in flying squirrel population fluctuations remains unclear. Low reproductive success of flying squirrels is related to low winter precipitation (Selonen et al. 2016; Selonen and Wistbacka 2016) and weather is spatially autocorrelated over large areas in Finland (> 1000 km for temperature; Uvo 2003; Zamorano et al. 2018). The spatial autocorrelation for precipitation is lower than that for temperature, but within our study area North Atlantic Oscillations create spatial autocorrelation also for winter precipitation (Uvo 2003). Thus, large-scale weather phenomena have the possibility to affect flying squirrel population fluctuations over large spatial scales. However, the weather determines also tree mast, which is spatially autocorrelated over large distances (Ranta et al. 2008; Zamarano et al. 2018). Although tree mast had no effect in the current analysis, its’ role for population dynamics of flying squirrels need further study (see below). Catkin availability is likely positively associated with temperature in summer when catkins develop (for birch Ranta et al. 2008, supplement Table S3). In the current analysis increased summer temperature (year_t-1_) had a slight positive effect on flying squirrel occurrence. Thus, if our catkin index could not perfectly predict the winter food availability for flying squirrels, the correlation between summer temperature at year_*t*−1_ and flying squirrel population growth rate might result from indirect effect of food availability. Alternatively, the positive correlation with summer temperature (year_*t*−1_) and growth rate in flying squirrel population size might be due to increased juvenile survival in warm weather (see, e.g. Studd et al. 2015).

Tree mast has clear impacts on the reproductive success of flying squirrels by advancing reproduction in spring and increasing production of second litters (Selonen et al. 2016; Selonen and Wistbacka 2016; Hoset et al. 2017). However, the positive effect of tree mast on reproduction may be counterbalanced by opposite effects of the environmental conditions in the following winter, because winters with good mast are normally followed by a winter with low food abundance (Gurnell 1996; Wauters et al. 2004, 2008; Selonen et al. 2015). These opposite environmental effects may explain the lack of correlation between tree mast and growth-rate estimate in the current study. It is also possible that the use of occupancy rate as a proxy for population abundance might mask some of the positive effects of food on population size, that is, if the occupancy rate did not perfectly reflect the changes in numbers of animals. For nest-box data, this seems unlikely, however, because flying squirrels are partly territorial and are usually found solitarily in nest-boxes (Selonen et al. 2014), and the occupancy rate of nest-boxes was relatively low in our data. In addition, it has been previously considered that occupancy of a typical Finnish forest patch by a flying squirrel, based on pellet surveys, reflects the occupancy of a single female territory (Hanski 2006). Consequently, there should be no major bias between occupancy rate and flying squirrel density in our data, except for the difference in detection probability due to difference in survey protocol (pellet survey vs nest-box occupancy, see methods).

Indices of abundance of main predators remained unrelated to population fluctuations of flying squirrels. Previous studies indicate that the presence of the main predator, the Ural owl, influences habitat use of flying squirrels (Byholm et al. 2012; Turkia et al. 2018b), but based on our results here and earlier studies (Koskimäki et al. 2014; Turkia et al. 2018b), it seems that the population-level effects of predators on flying squirrels remain minor (but see Jokinen et al. 2019). We did not find long-term monotonic trends in our data either, despite indications in other studies of declining trends for flying squirrel populations in recent decades (Selonen et al. 2010a; Koskimäki et al. 2014; Brommer et al. 2017). The interpretation of trends in the nest-box data may be problematic (McClure et al. 2017) because of a lag in occupancy and possible changes in unmonitored sites. Thus, our result of no trend should be taken cautiously, particularly because forest management inducing clear-cutting reduces forest habitat and natural cavities, and flying squirrels may need to move to occupy nest-boxes in the nearby forests.

Interestingly, we did not find increased spatial synchrony at distances that fall within the maximum dispersal distances of the species (9 km, Hanski and Selonen 2009; Selonen and Wistbacka 2017) or within distances where flying squirrel abundance forms spatially autocorrelated population patches (30 km, Remm et al. 2017). There was a possible decrease in synchrony with distances above 200 km (Fig. 3a), but this was related to higher levels of synchrony at the medium distances (20–200 km) and not at the shortest distances (< 20 km). Thus, within the timeframe of 3–25 years, the populations seem to have been strongly affected by very small-scale, local factors and demographic stochasticity that affect each site individually (see also, Brommer et al. 2017). Local changes in forest habitat quality, predator presence, nest-site availability and immigration patterns, are likely important factors determining the local occupancy patterns of flying squirrels. This may create metapopulation-like extinction–recolonisation dynamics on the flying squirrel-territory scale that creates asynchrony in local dynamics. Many earlier spatial synchrony studies have made dissimilar observations, perhaps because they have been carried out on abundant species (Paradis et al. 2000), whereas flying squirrels are relatively rare (Selonen and Mäkeläinen 2017). Nevertheless, the population dynamics of flying squirrels were also affected by long-term and large-scale factors synchronising population dynamics. Thus, the persistence of flying squirrel populations is not only determined locally.

We conclude that winter weather is an important factor behind population dynamics of flying squirrels. A change in winter weather also occurred during the observed periodic change in population fluctuations in flying squirrels. Thus, climate change may affect the degree of synchrony between populations, with potential implications for population dynamics (Post and Forchhammer 2004; Sheppard et al. 2015; Koenig and Liebhold 2016; Shestakova et al. 2016). Any changes in winter weather may create additional challenges for the species, which is already threatened by forest management (Jokinen et al. 2015). Understanding how weather shapes population dynamics will help predict population responses to climate change.

## Electronic supplementary material

Below is the link to the electronic supplementary material.

Supplementary material 1 (DOCX 308 kb)
