# Supplementary material for: Population fluctuations and spatial synchrony in an arboreal rodent
Source: Oecologia. 2019 Oct 30;191(4):861–71. doi: 10.1007/s00442-019-04537-3 (PMC6853850; doi:10.1007/s00442-019-04537-3)
Supplement: Supplementary file 1 — Supplementary material 1 (DOCX 308 kb) [file 442_2019_4537_MOESM1_ESM.docx]

Supplement

Figure S1. Time-series of flying squirrel occupancy in the 15 study sites. Before averaging (the uppermost bold black line), the values of all time-series were normalised to a mean of zero and a standard deviation of one: standard score z = (x - μ) / σ, where x is raw value of population abundance, μ is mean abundance overall years, and σ is standard deviation of abundance in the site. The vertical lines delineate the period of significant simultaneous cyclicity over all sites (2002–2006; see Fig. 1). On the diagram, the series of Luoto includes the subareas of Byrgmo and Eugmo combined, and Öskogen represents two series studied with different methods.

Figure S2. Mean growth rate and 95% confidence interval between the 15 study sites of flying squirrel occupancy in Finland. Circles indicate significant deviation from zero (orange positive and green negative).

Table S1. Population synchrony dependence on geographic distance in flying squirrel time-series during the years 2002–2006 (the time period indicated with wavelet analysis to have significant periodicity), and on the rest of the study years (1988–2001 and 2007–2015, when no significant periodicity was found). The estimated 95% confidence intervals (CI) of the average synchrony are based on 5,000 bootstrap permutations, the estimates of statistical significance of the GAMs (p) are based on comparisons of the empirical adjusted r² values with 5,000 randomly permuted 0-models (flat).

|  | Average synchrony, Pearson’s r | N_sites_ | N_pairs_ | GAM | | |  |
| --- | --- | --- | --- | --- | --- | --- | --- |
|  |  |  |  | Edf | F | r²_adjusted_ | p_5k permut._ |
| Abundance 2002–2006 | 0.29, CI: -0.001–0.56 | 12 | 128 | 1.82 | 5.20 | 0.07 | 0.30 |
| Growth Rate 2002–2006 | 0.32, CI: 0.04–0.74 | 12 | 128 | 1.90 | 8.59 | 0.11 | 0.13 |
| Abundance  1988–2001 & 2007–2015 | 0.13, CI: -0.07–0.35 | 16 | 156 | 1.71 | 1.15 | 0.01 | 0.52 |
| Growth rate  1988–2001 & 2007–2015 | 0.12, CI: -0.08–0.32 | 16 | 156 | 1.00 | 0.16 | -0.005 | 0.91 |

Table S2. Flying squirrel abundance modelled with events (flying squirrel observed); trial (total nest-boxes/sites surveyed) option and beta-binomial distribution in proc FMM in SAS 9.3. The Beta-binomial distribution was selected due to a slight over-dispersion in the data with binomial distribution. Site (n_sites_ = 9) was used as a class explanatory variable and year as a continuous variable to analyse population trends over time. Alder pollen estimates, predator pressure indices, and precipitation and temperature during different seasons were included in the model, separately for year_t_ and year_t-1_.

| Variable | average ± sd | estimate ± sd | z | p |
| --- | --- | --- | --- | --- |
| previous year* |  |  |  |  |
| alder pollen | 1450 ± 1700 in 1 m^3^ of air | -0.01 ± 0.023 | -0.49 | 0.62 |
| marten snow track | 1.1 ± 0.55 tracks in 24 h/10 km | 0.08 ± 0.07 | 1.09 | 0.28 |
| vole index | 9.4 ± 6.1 voles/100 trap nights | -0.006 ± 0.006 | -0.99 | 0.32 |
| winter rain | 41 ± 13 mm | 0.006 ± 0.002 | 3.47 | **0.0005** |
| winter snow cover | 24 ± 11 cm | -0.01 ± 0.005 | -1.9 | 0.07 |
| winter temperature | -5.1 ± 2.7 °C | -0.03 ± 0.02 | -1.46 | 0.14 |
| spring rain | 34 ± 13 mm | -0.004 ± 0.003 | -1.32 | 0.19 |
| spring temperature | 5.9 ± 1.4 °C | 0.002 ± 0.04 | 0.05 | 0.96 |
| summer rain | 67 ± 21 mm | 0.002 ± 0.002 | 0.77 | 0.44 |
| summer temperature | 15.2 ± 1.2 °C | 0.08 ± 0.04 | 2.01 | **0.04** |
| autumn rain | 59 ± 24 mm | -0.002 ± 0.002 | -1.48 | 0.14 |
| autumn temperature | 4.8 ± 1.6 °C | -0.003 ± 0.02 | -0.11 | 0.91 |
| current year* |  |  |  |  |
| alder pollen | 1450 ± 1700 in 1m^3^ of air | -0.009 ± 0.02 | -0.41 | 0.68 |
| marten snow track | 1.13 ± 0.55 tracks in 24h/10km | -0.03 ± 0.07 | -0.38 | 0.70 |
| vole index | 9.7 ± 6.3 voles/100 trap nights | -0.0002 ± 0.01 | -0.03 | 0.97 |
| winter rain | 40 ± 13 mm | 0.001 ± 0.002 | 0.42 | 0.67 |
| winter snow cover | 25 ± 12 cm | -0.006 ± 0.005 | -1.18 | 0.24 |
| winter temperature | -5.2 ± 2.8 °C | -0.03 ± 0.02 | -1.14 | 0.26 |

* Current and previous year values modelled in separate models. Climate variables are averages per month; alder estimate is the sum across the pollen season in spring.

Table S3. Spearman correlation coefficients for the weather variables and abundance indexes for voles, pine marten and alder pollen used in Table 2 of the main text. T = temperature, P = precipitation, t-1 = variable measured in previous year.

|  | **winterT** | **winterT**  **t-1** | **winterP** | **winterP**  **t-1** | **snow** | **snow**  **t-1** | **springT**  **t-1** | **springP**  **t-1** | **summerTt-1** | **summerP**  **t-1** | **autumnT**  **t-1** | **autumnP**  **t-1** | **vole** | **vole**  **t-1** | **marten** | **marten**  **t-1** | **alder** | **alder**  **t-1** |
| --- | --- | --- | --- | --- | --- | --- | --- | --- | --- | --- | --- | --- | --- | --- | --- | --- | --- | --- |
| **winterT** | 1,00 | 0,10 | 0,47 | 0,29 | -0,73 | -0,14 | -0,06 | -0,02 | 0,04 | -0,12 | 0,36 | 0,43 | 0,19 | 0,06 | 0,01 | 0,07 | 0,05 | -0,42 |
| **winterTt-1** | 0,10 | 1,00 | -0,06 | 0,41 | -0,24 | -0,75 | 0,16 | -0,16 | -0,22 | 0,42 | 0,07 | -0,08 | 0,10 | 0,14 | 0,14 | 0,00 | 0,03 | -0,01 |
| **winterP** | 0,47 | -0,06 | 1,00 | 0,26 | -0,21 | 0,05 | 0,05 | 0,12 | 0,01 | -0,03 | 0,31 | 0,39 | 0,32 | 0,01 | -0,05 | 0,20 | -0,05 | -0,36 |
| **winterPt-1** | 0,29 | 0,41 | 0,26 | 1,00 | -0,08 | -0,18 | 0,19 | -0,17 | 0,10 | 0,14 | 0,09 | 0,13 | 0,17 | 0,32 | 0,15 | 0,02 | -0,09 | -0,08 |
| **snow** | -0,73 | -0,24 | -0,21 | -0,08 | 1,00 | 0,41 | -0,03 | 0,05 | -0,18 | 0,10 | -0,25 | -0,26 | -0,15 | 0,04 | -0,01 | 0,07 | -0,09 | 0,32 |
| **snowt-1** | -0,14 | -0,75 | 0,05 | -0,18 | 0,41 | 1,00 | -0,12 | 0,20 | 0,24 | -0,34 | 0,14 | 0,09 | -0,03 | -0,09 | -0,13 | 0,07 | 0,10 | -0,03 |
| **springTt-1** | -0,06 | 0,16 | 0,05 | 0,19 | -0,03 | -0,12 | 1,00 | -0,13 | 0,35 | 0,23 | -0,05 | -0,12 | 0,24 | 0,28 | 0,09 | 0,08 | 0,04 | 0,28 |
| **springPt-1** | -0,02 | -0,16 | 0,12 | -0,17 | 0,05 | 0,20 | -0,13 | 1,00 | -0,03 | -0,08 | 0,25 | 0,32 | 0,25 | -0,45 | -0,20 | 0,00 | -0,28 | -0,05 |
| **summerTt-1** | 0,04 | -0,22 | 0,01 | 0,10 | -0,18 | 0,24 | 0,35 | -0,03 | 1,00 | -0,38 | 0,13 | 0,17 | -0,04 | 0,30 | 0,06 | 0,12 | 0,32 | -0,13 |
| **summerPt-1** | -0,12 | 0,42 | -0,03 | 0,14 | 0,10 | -0,34 | 0,23 | -0,08 | -0,38 | 1,00 | -0,07 | -0,21 | 0,31 | 0,10 | 0,14 | -0,22 | -0,19 | 0,09 |
| **autumnTt-1** | 0,36 | 0,07 | 0,31 | 0,09 | -0,25 | 0,14 | -0,05 | 0,25 | 0,13 | -0,07 | 1,00 | 0,18 | -0,07 | 0,04 | -0,10 | 0,23 | 0,06 | -0,33 |
| **autumnPt-1** | 0,43 | -0,08 | 0,39 | 0,13 | -0,26 | 0,09 | -0,12 | 0,32 | 0,17 | -0,21 | 0,18 | 1,00 | 0,12 | -0,09 | -0,06 | 0,10 | -0,05 | -0,28 |
| **vole** | 0,19 | 0,10 | 0,32 | 0,17 | -0,15 | -0,03 | 0,24 | 0,25 | -0,04 | 0,31 | -0,07 | 0,12 | 1,00 | -0,08 | 0,04 | -0,09 | -0,08 | -0,04 |
| **volet-1** | 0,06 | 0,14 | 0,01 | 0,32 | 0,04 | -0,09 | 0,28 | -0,45 | 0,30 | 0,10 | 0,04 | -0,09 | -0,08 | 1,00 | 0,13 | 0,10 | 0,30 | -0,05 |
| **marten** | 0,01 | 0,14 | -0,05 | 0,15 | -0,01 | -0,13 | 0,09 | -0,20 | 0,06 | 0,14 | -0,10 | -0,06 | 0,04 | 0,13 | 1,00 | 0,18 | -0,11 | 0,16 |
| **martent-1** | 0,07 | 0,00 | 0,20 | 0,02 | 0,07 | 0,07 | 0,08 | 0,00 | 0,12 | -0,22 | 0,23 | 0,10 | -0,09 | 0,10 | 0,18 | 1,00 | 0,19 | -0,14 |
| **alder** | 0,05 | 0,03 | -0,05 | -0,09 | -0,09 | 0,10 | 0,04 | -0,28 | 0,32 | -0,19 | 0,06 | -0,05 | -0,08 | 0,30 | -0,11 | 0,19 | 1,00 | -0,38 |
| **aldert-1** | -0,42 | -0,01 | -0,36 | -0,08 | 0,32 | -0,03 | 0,28 | -0,05 | -0,13 | 0,09 | -0,33 | -0,28 | -0,04 | -0,05 | 0,16 | -0,14 | -0,38 | 1,00 |
